# Sediment resuspension due to internal solitary waves of elevation in the Messina Strait (Mediterranean Sea)

**DOI:** 10.1038/s41598-023-33704-z

**Published:** 2023-05-04

**Authors:** Giovanni La Forgia, Riccardo Droghei, Martina Pierdomenico, Pierpaolo Falco, Eleonora Martorelli, Alessandro Bergamasco, Andrea Bergamasco, Federico Falcini

**Affiliations:** 1 University of Cassino and Southern Lazio, Cassino, Italy; 2 Liceo Scientifico Francesco Severi, Frosinone, Italy; 3 Institute for the Study of Anthropic Impact and Sustainability in the Marine Environment, Consiglio Nazionale delle Ricerche (IAS-CNR), Rome, Italy; 4 Marche Polytechnic University, DISVA, Ancona, Italy; 5 Institute of Environmental Geology and Geoengineering, Consiglio Nazionale delle Ricerche (IGAG-CNR), Rome, Italy; 6 Institute of Marine Sciences, Consiglio Nazionale delle Ricerche (ISMAR-CNR), Venice, Italy; 7 Institute of Marine Sciences, Consiglio Nazionale delle Ricerche (ISMAR-CNR), Rome, Italy

**Keywords:** Ocean sciences, Physical oceanography

## Abstract

By combining real-field observations and theoretical predictions, we describe role and relationships among north-propagating internal solitary waves (ISWs) generated by tidal currents in the Messina Strait (Mediterranean Sea), buoyancy deformation, sediment resuspension, and mixing effects. In particular, our results show that the presence of ISWs traveling along the Gioia Basin (north of the Strait) is not strictly related to seasonality. During winter, when the remote observation of ISWs from satellite is particularly rare due to the weak water column stratification, we observe elevation-type ISWs from hydrographic data. This finding reveals a different scenario with respect to the summer one, when the high stratified water column gives rise to depression-type north-propagating ISWs and the subsequent sea surface manifestations, detectable from satellite imagery. Moreover, our beam transmission observations and theoretical predictions of the induced near-bottom horizontal velocity suggest that these elevation-type ISWs induce sediment resuspension over the seafloor, as well as mixing effects as they break on the frontal slope nearby Capo Vaticano.

## Introduction

**Figure 1:**
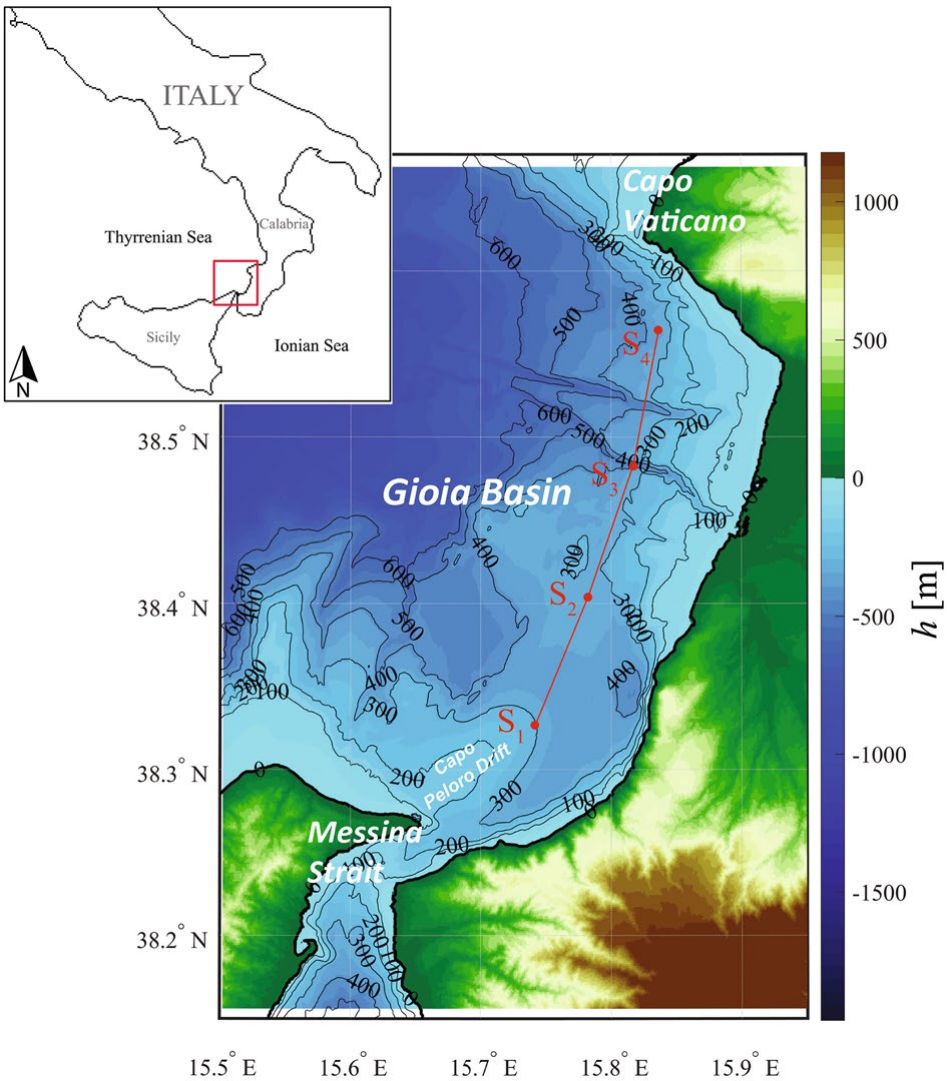
Map of the Gioia Basin with the location of measurement stations $S_{1}$–$S_{4}$. Map is generated by using MATLAB 7.1 http://uk.mathworks.com/products/matlab.

The Messina Strait (Fig. 1), separating the Italian peninsula from Sicily Island, constitutes a natural element of connection between the Tyrrhenian Sea and the Ionian Sea, which are located respectively in the western and eastern basin of the Mediterranean Sea. The Strait constitutes an underwater constraint for the waters that flow within it. This leads to the formation of a vast oceanographic phenomenology. In particular, the presence of harmonic (semi-diurnal) tidal motions between the Ionian and Tyrrhenian sub-basins induces hydrographically controlled tidal currents and, subsequently, turbulent flows and nonlinear wave propagation^1–4^.

Within the strait there is a high tidal gradient of the sea level, due to a phase opposition ($\sim$ 5 h) between the Tyrrhenian Sea and the Ionian Sea^5^ (Fig. 1); at the sill of the strait, indeed, there is one of the secondary amphidromic points of the Mediterranean Sea. Due to this phase opposition and to topographic constraints, the marine currents within the strait reach values of $\sim$3 m/s^6–8^. A northward current is recorded around the sill region (i.e., the so-called *Rema Montante*) during the 3–9 h following the upper or lower southern passage of the Moon, while the flow is reversed (i.e., the southward current named *Rema Scendente*) during 3–9 h following the previous stream. These currents slow down significantly in the period between the two maximum flows (i.e., the *Stanca* weak, residual current).

**Figure 2:**
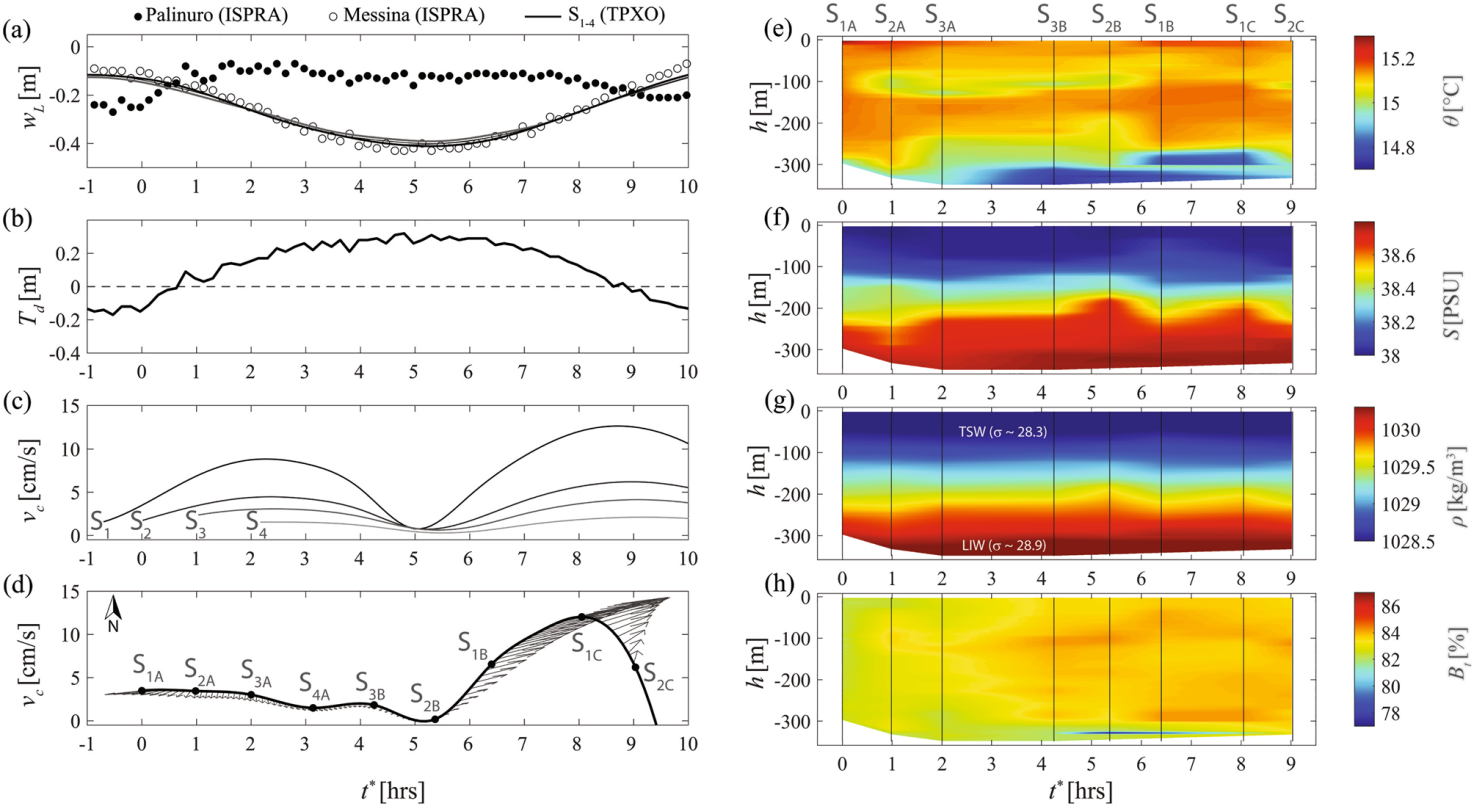
(**a**) ISPRA sea level elevations ($w_L$) measured South (solid dots, Messina, Sicily) and North (empty dots, Palinuro, Calabria) of the Messina Strait; predictions of sea level elevations from the TPXO global tidal model at stations $S_{1-4}$ (lines). (**b**) Tidal displacement ($T_d$) obtained as the difference between ISPRA sea surface elevations measured and South the Messina Strait. (**c**) Temporal evolution of the barotropic current velocities ($v_c$) predicted from TPXO at station $S_{1-4}$ during the oceanographic cruise; (**d**) Barotropic current velocities at each station during the hydrographic measurements (stick diagrams are also shown). (**e**–**h**) Time evolution of the associated oceanographic data at stations $S_{1-3}$ collected during the RITMARE 2016 cruise in February 2016 (i.e., interpolated (e) potential temperature ($\theta$), (**f**) salinity (*S*), (**g**) density ($\rho$) and (**h**) beam transmission ($B_t$); in these panels we did not considered $S_{4}$ station in order to exclude lateral boundary effects due to ISW breaking; panel (**g**) also indicates the two main water masses that characterize the Gioia Basin, i.e., the TSW ($\rho \sim 1028.4 \ kg/m^{3}$, $\sigma \sim 28.3$) and the LIW ($\rho \sim 1030.3 \ kg/m^{3}$, $\sigma \sim 28.9$)^2^; for each hydrographic quantity, we derived time interpolated values by adopting a cubic convolution. Time ($t^{\star }$) indicates the time since the beginning of the hydrographic data acquisition in $S_{1A}$. Plots are generated by using MATLAB 7.1 http://uk.mathworks.com/products/matlab.

**Figure 3:**
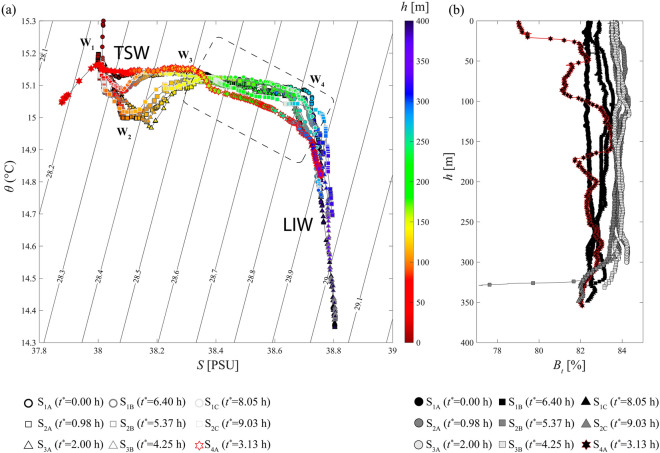
(**a**) Potential temperature-Salinity diagram for the 9 CTD measurements and (**b**) the associate beam transmission profiles. Color coding indicates depth (m) and dashed lines show isopycnals with contours of potential density anomaly^28^ ($kg/m^3$). Panel (**a**) also indicates the two main water masses that characterize the Gioia Basin, i.e., the TSW ($\sigma \sim 28.3$, $\theta \sim 15.2^\circ$C , $S\sim 38.0$ psu) and the LIW ($\sigma \sim 28.9, \theta \sim 14.5^\circ$C, $S\sim 38.8$ psu)^2^. Plots are generated by using MATLAB 7.1 http://uk.mathworks.com/products/matlab.

From a hydrological point of view, two different water masses are affected by the tidal effects occurring in the Messina Strait^2^ (Figs. 1, 2g, 3a): the *Tyrrhenian Surface Water* (TSW), which covers the sea surface layer of the Tyrrhenian Sea, and the denser *Ionian Intermediate Waters* (IIW), flowing from the Ionian Sea at intermediate depths as a branch of the Levantine Intermediate Water (LIW; hereafter we will refer to the LIW also for the Messina Strait, in accordance with the previous literature). Within the strait, these two waters are separated by an interface, located about 150 m deep^6^. From the baroclinic compensation of the tidal motion^2,9^ it results that the *Rema Montante* represents the direct northward flow of the LIW, which gives rise to an injection of Ionian water into the Tyrrhenian Sea, while the *Rema Scendente*, at the sea surface, implies a southward flow of the lighter TSW into the Ionian Sea^5,10^. The sill of the Strait is, in fact alternately, entirely occupied by these two water masses. This implies semi-diurnal oscillations of the interface between the LIW and the TSW of $\sim$100 m, which reaches, with semi-diurnal periodicity, both the seabed and the surface of the sea^11^.

The tidal pattern we described, along with the topographic constraint of the Strait gives rise to internal solitary waves (ISWs)^1,3,12–16^. These nonlinear waves oscillate along the interface between LIW and TSW, propagating at $\sim$1 m/s from the sill of the MS both northward and southward, alternately (approximately with a 6-h period). Sea surface manifestation of ISWs in the MS are frequently observed from Synthetic Aperture Radar (SAR) satellite images^1,3,17,18^. In the northern side of the strait (i.e., the Gioia Basin; Fig. 1), these sea surface markers are particularly visible during spring and summer seasons, i.e. when a strong seasonal pycnocline is known to be present, due to the water column stratification^12,13,19,20^.

Over the Gioia Basin, ISWs propagating northward significantly reshape the seafloor as suggested by two fields of northward migrating sand waves over the southern and northern tip of the Capo Peloro contourite drift (CPD in Fig. 1), likely related to ISWs action^4,11^. Furthermore, contourite deposits linked to the LIW action were recognized off Palmi Ridge^21^ and south of Capo Vaticano^22^. In particular, south of Capo Vaticano, contourites likely develop by sediment resuspension induced by the increase of bed shear stress that is generated by ISWs breaking^22^. In addition, breaking of ISWs has been considered as a potential trigger for slope instability and turbidity currents south of Capo Vaticano^16^. Based on grain size analyses and multivariate statistics on grab samples, sediment types characterzing the morpho-sedimentary structures of the area were recognized as muddy fine sand, with local presence of gravel and gravelly sediment within canyons and off promontories^21^.

Despite multiple indications of the relation between ISWs and morpho-sedimentary structures in the Gioia Basin^11,23,24^, as well as the extensive hydrographic analysis that captured ISWs in the MS^12^, a direct observation of ISW-induced sediment resuspension is still missing. To fill this gap, here we aim to understand and quantitatively describe role and relationships among north-propagating ISW, buoyancy deformation, sediment resuspension, mixing effects at the bottom layer and at northern boundary of the Gioia Basin, i.e., Capo Vaticano (Fig. 1). To this purpose we combine hydrographic, in situ observation with tidal model outputs in order to relate spatial and temporal variation of the buoyancy with bottom sediment resuspension, observed from transmissometer.

## Data and methods

### Hydrographic data

During the oceanographic cruise RITMARE 2016, we collected hydrographic data in the Gioia Basin, along a SW–NE-oriented transect that follows the expected northward propagating ISWs path (Fig. 1). Along this transect, for about 10 h (i.e., between 20:32 21 February 2016 and 05:34 22 February 2016, UTC+1), we sampled at each station in the following order (Fig. 2; Table 1): northward, from $S_{1}$ to $S_{4}$ (herein $S_{1A}$, $S_{2A}$, $S_{3A}$, $S_{4A}$); then, southward, from $S_{4}$ to $S_{1}$ (herein $S_{3B}$, $S_{2B}$, $S_{1B}$); northward, from $S_{1}$ to $S_{2}$ (herein $S_{1C}$, $S_{2C}$). For each station, we collected hydrographic data (conductivity-temperature-depth, CTD) and beam transmission along the water column. Large beam transmission is associated to low water turbidity while small values of beam transmission may indicate the occurrence of turbulence at the bottom layer, which may give rise to sediment resuspension. Since we collected hydrographic data in multiple locations at successive times, our measurements are associated to different hydrodynamic conditions.

**Table 1:** CTD measurements: stations names and locations in geographic coordinates (WGS84), date and time of collected data.

Station name	Date	Local time	Longitude ($^{\circ }$)	Latitude ($^{\circ }$)
$S_{1A}$	02/21/2016	20:32	38.32682	15.74168
$S_{2A}$	02/21/2016	21:31	38.40368	15.7823
$S_{3A}$	02/21/2016	22:32	38.48246	15.81724
$S_{4}$	02/21/2016	23:40	38.56436	15.83648
$S_{3B}$	02/22/2016	00:47	38.48246	15.81724
$S_{2B}$	02/22/2016	01:54	38.40368	15.7823
$S_{1B}$	02/22/2016	02:56	38.32682	15.74168
$S_{1C}$	02/22/2016	4:35	38.32674	15.74172
$S_{2C}$	02/22/2016	5:34	38.40370	15.78238

### Tidal data

At each station, we obtained tidal elevation (respect to the Mean Sea Level) and barotropic, tidal velocities from the Oregon State University TOPEX/Poseidon Global Inverse Solution tidal model^25^ (TPXO). TPXO uses satellite altimetry to constrain solutions to the shallow water Laplace tidal equations on a 1/30$^{\circ }$ bathymetric grid. Time–varying velocities and free-surface elevations were reconstructed from 12 tidal constituents (i.e., $M_2$, $S_2$, $K_1$, $O_1$, $N_2$, $P_1$, $K_2$, $Q_1$, $2N_2$, $M_4$, $MS_4$, $MN_4$). We then compared the TPXO model predictions with tidal elevations collected by the Italian National Institute for Environmental Protection and Research (ISPRA). In particular, we considered the time of the first measurement in section $S_{1A}$ as reference time, i.e. $t_0=20{:}32$ (local time) on 21 February 2016. Once the TPXO data are validated with the ISPRA in situ data, we solely made use of the TPXO data for our analysis. Thus, we computed the relative time evolution as $t^{\star }=t-t_0$.

## Hydrographic analysis

In situ tidal displacement measured North (Palinuro, Calabria) and South (Messina, Sicily) of the Strait of Messina shows the phase opposition of the semidiurnal tides (Fig. 2a,b). TPXO predictions are in good agreement with the corresponding measured values (see empty dots and lines in Fig. 2a). Both datasets show that the semi-diurnal southward flux of TSW (i.e., the *rema scendente*) is expected to start around $t^{\star }=1$ hr (Fig. 2c,d). At that time, the magnitude of the barotropic tidal velocity shows, indeed, increasing values (Fig. 2c), although, our measurements are collected by moving away from the strait (Fig. 2d); the current velocities rapidly decrease^23^ when moving northward (Fig. 2c,d). This is due to the locations of the stations: the more the distance from the strait increases, the more the speed peak is delayed in time (Fig. 2c). The smallest current velocities are observed all over the basin at approximately $t^{\star }=5$ hr, as the tidal displacement occurring at the sill region reaches its maximum (Fig. 2a,b).

Remarking that $S_{4}$ is located too close to the northern topographic constraint of the Gioia Basin (i.e., Capo Vaticano frontal slope), for a better understanding of the hydrodynamic features occurring over the central portion of the Gioia Basin and thus to avoid observations of lateral boundary effects (such as wave breaking effects), here we consider hydrographic data collected at Stations $S_{1}$, $S_{2}$, and $S_{3}$. From these hydrographic data we relate the temporal evolution of in situ potential temperature, salinity, density, and beam transmission with the tidal regime of the basin (Fig. 2e–h; CTD casts were time-interpolated by adopting a cubic convolution to provide a spatio-temporal evolution of the hydrographic characteristics of the water column). For $t^{\star }<2$ hrs, i.e., moving from $S_{1A}$ to $S_{3A}$, we observe the later stages of the *rema scendente*, characterized by low, SW-oriented, current velocities (Fig. 2d). Accordingly, hydrographic measurements show similar vertical profiles from $S_{1A}$ to $S_{3A}$, characterized by the presence of two pycnoclines at $\sim$100 m and $\sim$250 m depth (Fig. 2g). Moreover, at $S_{1A}$ and $S_{2A}$ we notice a homogeneous warm water, till 300 m depth, that tends to fill the entire column in the $S_{1}-S_{2}$ area. This may be associated to TSW southward flow (Fig. 2e,f). Interestingly, we also observe an intrusion of a cold water at $\sim$100 m depth in $S_{2}-S_{3}$ region (Fig. 2e), which is associated with a change of beam transmission at the same depth (Fig. 2g and Fig. S1 in supplementary information).

At $t^{\star }=2.5$ hrs, as the *rema scendente* decreases, the vessel was too far from the Strait (i.e., between $S_{3}$ and $S_{4}$) and thus it could not be affected by the tidal flow. Indeed, for $3<t^{\star }<5$ hrs the tidal current predicted at stations $S_{4A}$, $S_{3B}$ and $S_{2B}$ shows a weak SW current. In particular, at $t^{\star }=4.2$ hrs (station $S_{3B}$), we observed a cold and salty water along the last 60 m of the water column, likely associated to the readjustment of the density profile^2^, where the TSW no longer fills the whole water column and the LIW, debouching from the Strait during the *rema montante*, remains in the bottom layer of the basin (Fig. 2e,f).

For $t^{\star }>4.2$ hrs, despite the phase of a low barotropic tidal velocity (Fig. 2d), displacements of both pycnoclines are clearly observed (Fig. 2g). This corresponds with minimum values of beam transmission close to the seafloor (i.e., the last 10 m) in section $S_{2B}-S_{3B}$ (Fig. 2h). This evidence suggests the presence of turbid layer, presumably due to sediment resuspension induced by the propagation of the ISW^4,26,27^.

For $t^{\star }>5$ hrs we observe the continuous increase of the NE tidal current (Fig. 2d) during the measurements in stations $S_{2B}$, $S_{1B}$, $S_{1C}$ and $S_{2C}$ (Fig. 2d). This pattern marks the presence of *rema montante* in this region. At this time frame, we found high turbidity in $S_{1B}$, which may mark a remaining resuspension of sediment due to the ISW passage, previously occurred at this station around $t^{\star }\sim 2$ hrs. Unlikely, this resuspension might be also due to the tidal current (i.e., *rema montante*) that, in $S_{1B}$ reaches $\sim$ 10 cm/s (Fig. 2c).

$\theta -S$ plots for at $S_{2}$ and $S_{3}$, (Fig. 3) show four hydrographic features, i.e., $W_1$ (15.3 $^{\circ }$C, 38 psu, $\sigma$=28.25), $W_2$ (15 $^{\circ }$C, 38.1 psu, $\sigma$=28.35), $W_3$ (15.1 $^{\circ }$C, 38.3 psu, $\sigma$=28.50) and $W_4$ (15.1 $^{\circ }$C, 38.7 psu, $\sigma$=28.80). $W_1$ marks the upper mixed layer within the Gioia Basin, whose thickness is approximately 80 m (Fig. 3); $W_2$ marks the presence of a halocline at h $\sim$120 m (Fig. 3); this hydrographic region marks the presence of the TSW^2^. Between $W_2$ and $W_3$, a homogeneous layer precedes a well-stratified region, observed from $W_3$ to $W_4$, characterized by an almost linear decrease of the salinity. At h $\sim$260 m (below $W_4$), a lower thermocline separates the previous region by the bottom layer, characterized by even colder water mass, i.e., the LIW^2^.

$\theta -S$ data for station $S_{4A}$ show a completely different trend with respect to the other stations. Here we observe the presence of an upper mixed layer (above $W_1$), and the subsequent increase of salinity along 150 m of the water column (from $W_1$ to $W_3$) while temperature remains constant ($\sim$15.15 $^{\circ }$C). Such a singular behavior highlights that in this S4 region, differently from $S_{1}$ to $S_{3}$, the upper (sub-surface) portion of the water column is stretched by mixing phenomena occurring at intermediate layer^16,26^. Between 150–200 m depth a step-like behavior in the $\theta -S$ diagram reveals the presence of a thick pycnocline (thicker than the those we observe in the other regions), separating the above hydrologic behavior from a second colder and saltier water. We also observe that the deep water mass (from $W_3$ to $W_4$) has a lower temperature than the one observed at $S_{1}$ and $S_{3}$.

## The role of ISWs

**Figure 4:**
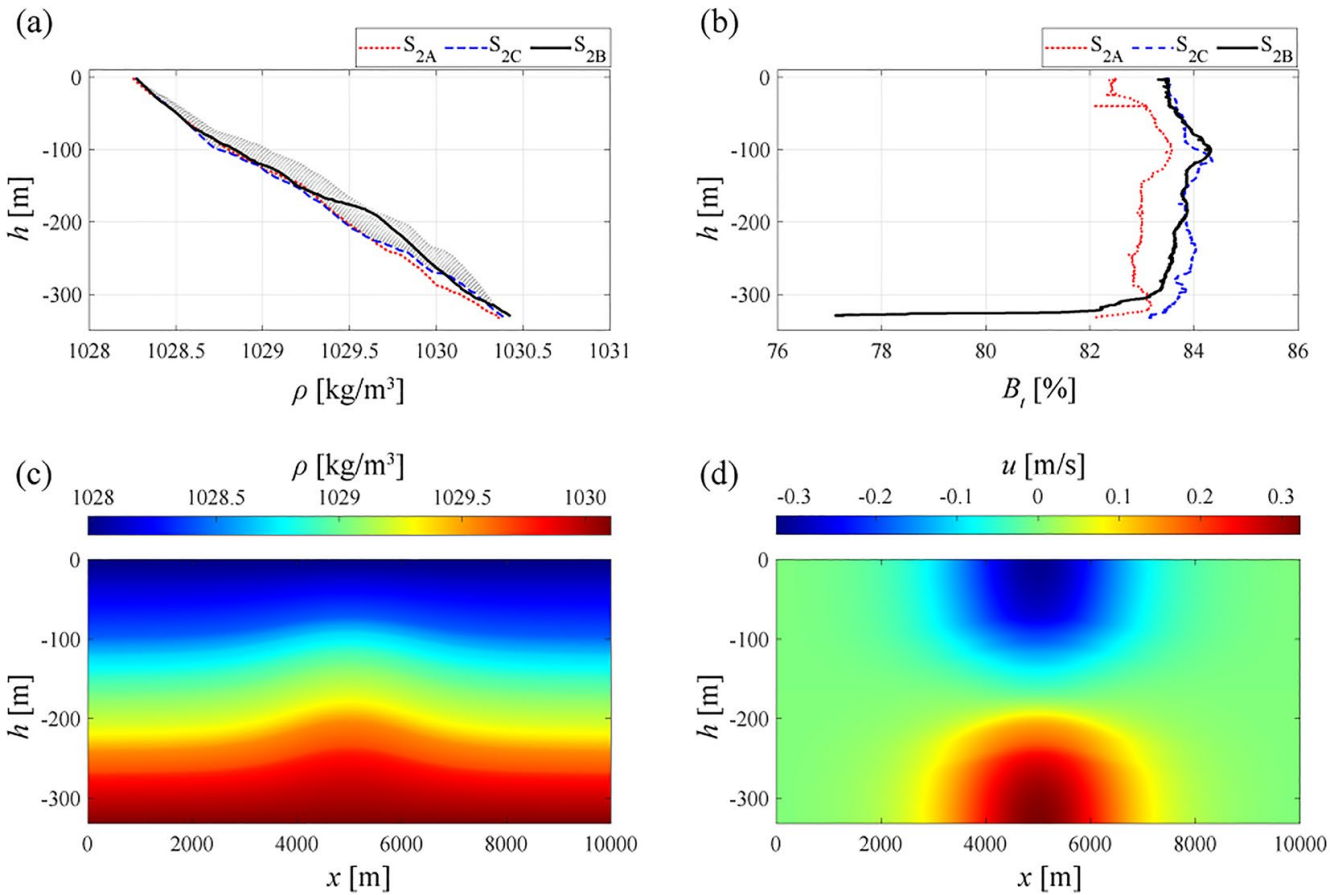
(**a**) Density profiles and (**b**) beam transmission distribution for station $S_{2}$ during three successive measurement times (i.e. $S_{2A}$, $S_{2B}$, and $S_{2C}$); gray-dashed area refers to density profiles predicted by the DJL model along the *x*-axis during the ISW passage. (**c**) Density and (**d**) horizontal velocity distribution predicted by the DJL model over a distance that is comparable with the Gioia Basin longitudinal dimensions. Set-up, boundary and initial conditions of the model are taken from the hydrographic conditions we show in Fig. 2. Plots are generated by using MATLAB 7.1 http://uk.mathworks.com/products/matlab.

Thickening of the pycnocline we observe at $S_{4}$ (between 150 and 200 m) and the presence of a colder and saltier water in this station (from $W_3$ to $W_4$ in Fig. 3) confirm the presence of wave-breaking phenomena that mix TSW and LIW in the northern part of the Gioia Basin^20,29^ (Fig. 3a). This is also confirmed by beam transmission profiles (Fig. 3b): constant $B_t$ values at stations $S_{1}$ and $S_{3}$ up to 300 m depth and a highly fluctuating $B_t$ profile in $S_{4}$ along almost all the water column, with a mean $B_t$ value lower than those at $S_{1}$ and $S_{3}$. These oscillations can be attributed to turbulent mixing and the subsequent sediment resuspention and nepheloid layers formation due to wave-breaking processes^16^.

CTD measurements collected at station $S_{2}$ show two main evidences: (i) an upward displacement of the isopycnals for the cast $S_{2B}$, measured at $t^{\star }=5.37$ hrs (i.e., station $S_{2B}$), with respect to previous and following profile measured at the same station, i.e., $S_{2A}$ and $S_{2C}$ (Fig. 4a); (ii) an anomalous, low values of beam transmission ($B_t$) at the bottom layer, along the last 30 m of the water column (Fig. 4b). We note that, at this station, TPXO local predictions show a very low tidal velocity (Fig. 2c), that is, neither near bed sediment resuspension highlighted by beam transmission nor density variations could be directly related to tidal barotropic currents. For these reasons, we argue that the observed anomalies are likely due to the northward propagation of ISWs, generated nearby the sill region in conjunction with the spread of the withdrawal of *rema scendente*. Indeed, in previous experimental investigations^4,12^, ISW in the Gioia Basin were found to induce near bed velocity greater than 30 cm/s, a value that suggests resuspension of sandy sediment and that was theoretically confirmed^30^.

To theoretically confirm this hypothesis, we consider the Dubreil–Jacotin–Long (DJL), fully nonlinear and strongly dispersive model^31,32^. The DJL model is equivalent to the full set of the stratified Euler equations for a reference frame moving with the wave, providing the exact ISW solutions for inviscid conditions, under the Boussinesq approximation. For a channel of finite depth with a background current *U*(*y*), the DJL equation for the channel of finite depth *H* is given by:

1 $$\begin{aligned} \begin{aligned} {\nabla }^2&\eta +\frac{N^2(z-\eta )}{{c_w}^2}\eta =0; \\&\eta =0 \; at \; z=0, H; \\&\eta \xrightarrow []{}0 \; as \; z\xrightarrow []{}\pm \infty . \end{aligned}\end{aligned}$$

where $\eta = \eta (x, z)$ represents the vertical displacement of the streamline passing through (*x*, *z*) relative to its far-field height, $c_w$ is the ISW celerity in a channel of finite depth *H*, $N(z)^{2} = g d\rho (z) / dz$ is the square root of the buoyancy frequency, *g* is the gravitational acceleration and $\rho (z)$ represents the undisturbed, dimensionless density profile.

We used the Matlab version of the DJL code developed by Dunphy et al.^33^, in order to apply the numerical solution for solitary waves in continuously stratified flows to the case of the Strait of Messina^34–36^. The code seeks a solution that, iteratively, minimizes the kinetic energy, for a specified value of the wave available potential energy (*APE*). The latter quantifies the system potential energy that can be converted into kinetic energy, and, scaled by $\rho _0gH$, it is defined as:

2 $$\begin{aligned} APE(\eta )=\frac{1}{H}\int _{0}^{H}\int _{-\infty }^{+\infty }[{\bar{\rho }}(z-\eta )-{\bar{\rho }}(z-s)dsdxdz]. \end{aligned}$$

In equation (2) we assume that the density profiles measured at $S_{2A}$ and $S_{2C}$ represent the undisturbed conditions that define the density distribution before and after the ISWs passage. To obtain a numerical solution from the DJL model we set two main quantities: the wave celerity $c_w$ and the solitary wave *APE*. In order to define $c_w$, we consider that northward propagating ISWs are generated nearby the sill region during the withdrawal of *rema montante*^3,12,19^ (i.e. for $t^{\star } = 0$ in Fig. 2a,b). Thus, we estimate the minimum celerity that a single ISW would have to reach station $S_{2B}$ at $t^{\star }=5.37$, propagating from the sill region at $t^{\star }=0$. Associated to a linear distance of about 17070 m separating the sill from station $S_{2B}$, a wave celerity of $c_w$ = 0.883 m/s is expected. By setting the wave *APE* equal to $2x10^4$ $m^4s^{-2}$, the DJL model predicts a maximum pycnoclines displacement with the same order of magnitude of the ones resulting from CTD measurements (i.e. about 50 m at the wave crest in Fig. 4c). Density profiles associated to the presence of the ISW smulated by the DJL model (see gray-dashed area in Fig. 4a) appear in good agreement with the vertical density distribution measured in station $S_{2B}$ (solid black line in Fig. 4a). The numerical solution of the DJL model suggests that tidally generated ISWs of elevation can propagate northward trough the considered density distribution. They are expected to be characterized by a relatively large wavelength (approximately 6 km, Fig. 4c), i.e. one order of magnitude larger than the one measured for ISWs of depression during summer^13^. Velocity distribution predicted by the DJL model show a 960 m long and 40 m high region where the induced near-bed horizontal velocities assume values larger than 0.3 m/s (Fig. 4d).

Many studies analyzed mechanisms of instability that leads to sediment resuspension due to ISW-BBL interaction^37^. For the typical oceanic density stratification, consisting of a thin upper mixed layer above a thicker benthic zone, ISWs are waves of depression. For this reason, only few studies investigated sediment resuspension induced by elevation-type ISWs^37^.

From theoretical considerations on real field observations, Bogucki et al.^38^ argued that ISWs of elevation give rise to favorable conditions for resuspension in response to a global instability occurring near the front of the wave. Numerical study of Moum et al.^39^ confirmed this hypothesis: a near-bottom peak in turbulent dissipation occurs at the front of their shoaling waves of elevation.

Laboratory experiments performed by La Forgia et al.^27^ showed that the velocity field induced by surface solitary waves, similar to the one associated to elevation-type ISWs, induce sediment transport when acting on a flat, mobile sandy bed. It is worth noting that free stream velocities measured from their laboratory experiments assumed values very similar to those predicted by the DJL model in the present study.

## Discussion and conclusions

From the synergy among CTD and beam transmission in situ measurements, knowledge of tidal currents estimated from TOPEX/Poseidon Global Inverse Solution, and the Dubreil–Jacotin–Long model we investigated and directly recognize the ability of northward propagating ISWs from the Messina Strait to resuspend sediments in the Gioia Basin.

Our analysis focused on hydrographic data collected during the winter season, i.e., for water column stratification that is considerably weaker than those occurring in the summer. This implies that the ISW we analyzed could not show any sea surface manifestations during its propagation and, thus, could not be captured by SAR imagery^40^. Despite the lack of sea surface markers, we proved that these elevation-type, northward propagating ISWs are active in the Gioia Basin. Indeed, perturbation of vertical density profile observed from CTD agreed with the ISWs features derived from the DJL model.

The DJL near-bottom horizontal velocity results to be larger than 0.3 m/s. This value can be linked to lowest values of beam transmissions registered at stations where we observed pycnoclines displacements, i.e., $S_{2B}$. Such a result and, in particular, the synergy of in situ measurements with the DJL modelling, counter proved the fact that northward (southward) propagating ISWs from the Messina Strait are triggered by the withdrawal the *rema montante* (*rema scendente*).

We also found that ISWs traveling along the Gioia Basin are not strictly related to seasonality. Despite the lack of ISW observation from sea surface effects, we found that winter stratification gives rise to elevation-type ISWs (while depression-type ISWs are usually observed during spring-summer measurements from satellite^12^). The sill located at the Messina Strait behaves as a bathymetric constraint, leading the semi-diurnal nortward propagating tidal flow to excite iso-density lines displacements within the Gioia Basin. Due to the relatively deep pycnoclines location, as revealed from CTD measurements, elevation-type ISWs are expected to form and propagate northward^41^.

In particular, we found that these waves are able to induce an excess of bed shear stress that reshapes the seafloor by sediment resuspension from stations $S_{1}$ to $S_{3}$.

The persistency (during winter and summer) of the interaction between ISWs and the frontal slope of Capo Vaticano, represents a stationary characteristics for dense water formation in the Gioia Basin^11,16,24^. Moreover, the presence of a seasonal ISWs of elevation might reinforce the hypothesis of northward migrating dune fields in the Gioia Basin^4,11^. Our analysis also suggests that ISWs break as they interact with the shelf topography nearby Capo Vaticano, causing mixing and resuspension of seafloor sediment.

Our analysis confirms that the Messina Strait and, in particular, the Gioia Basin are very peculiar areas, representing a unique observatory for turbulent phenomena and their related impacts on morpho-sedimentary and ecological systems^4,11,16,24,42,43^. Besides the relation between bed shear stress and bed forms occurring in the Gioia Basin, sediment resuspension we observe may open to the hypothesis of ISW-induced triggering of downslope sediment-gravity currents, even in the winter season, when the poorly-stratified water column conditions would inhibit downwelling processes^42^. These hypothesized sediment-gravity currents may contribute to the shallow-to-deep water exchanges that are well documented in the Gioia Basin^22,24,44^. Finally, the observation of mixing effects due to ISW breaking off Capo Vaticano, nearby station $S_{4}$, fits with the high phytoplankton productivity and biodiversity that characterize this region, also during the winter season^42,43^. Besides, shoaling and breaking processes associated to elevation-type ISWs crucially differ from those observed for waves of depression^45^. This represents an open issue that deserves further investigation that could help to clarify the role of ISWs occurring during the cold season in modifying the density distribution within the water column.

## Supplementary Information

Supplementary Figure 1.

## Data Availability

The datasets used and/or analysed during this study are available from the corresponding author on reasonable request.
