# Supplementary material for: Sediment resuspension due to internal solitary waves of elevation in the Messina Strait (Mediterranean Sea)
Source: Sci Rep. 2023 May 4;13:7229. doi: 10.1038/s41598-023-33704-z (PMC10160082; doi:10.1038/s41598-023-33704-z)
Supplement: Supplementary file 1 — Supplementary Figure 1. [file 41598_2023_33704_MOESM1_ESM.pdf]

Supplementary Information

Sediment resuspension due to internal solitary waves of elevation in the Messina Strait (Mediterranean Sea)

Giovanni La Forgia[1], Riccardo Droghei[2], Martina Pierdomenico[3], Pierpaolo Falco[4], Eleonora Martorelli[5], Alessandro Bergamasco[6], Andrea Bergamasco[6], and Federico Falcini*[6]

[1]University of Cassino and Southern Lazio, Cassino (FR), Italy
[2]Liceo Scientifico Francesco Severi, Frosinone, Italy
[3]Institute for the Study of Anthropic Impact and Sustainability in the Marine Environment, Consiglio Nazionale delle Ricerche (IAS-CNR), Rome, Italy
[4]Università Politecnica delle Marche, DISVA, Ancona, Italy
[5]Istituto di Geologia Ambientale e Geoingegneria, Consiglio Nazionale delle Ricerche (IGAG-CNR), Rome, Italy
[6]Institute of Marine Sciences, Consiglio Nazionale delle Ricerche (ISMAR-CNR), Rome, Italy
[*]corresponding author, federico.falcini@cnr.it

Supplementary Figure 1

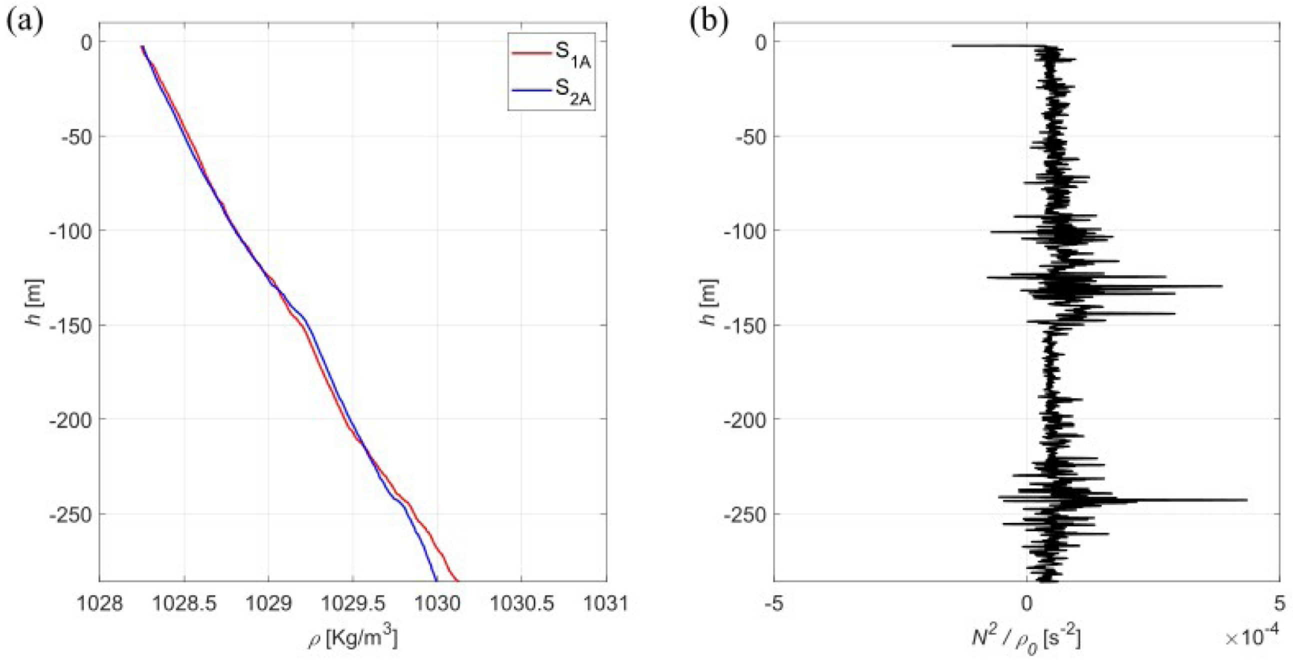

Figure 1. Undisturbed condition: (a) density profiles associated to stations S_{1A} and S_{2A}; (b) corresponding vertical distribution of the averaged buoyancy frequency N^2 divided by the reference density $\rho_0 = 1000$ kg/m^3.
